# Expression patterns and prognostic value of m^6^A RNA methylation regulators in adrenocortical carcinoma

**DOI:** 10.1097/MD.0000000000025031

**Published:** 2021-03-12

**Authors:** Yang Fu, Shanshan Sun, Jianbin Bi, Chuize Kong, Lei Yin

**Affiliations:** a Department of Urology; b Department of Pharmacy, The First Hospital of China Medical University, Shenyang, PR China.

**Keywords:** adrenocortical carcinoma, cluster analysis, m^6^A, prognosis, risk score

## Abstract

Adrenocortical carcinoma (ACC) is considered a rare cancer with poor prognosis. We used public datasets from The Cancer Genome Atlas (TCGA) and Genotype-Tissue Expression (GTEx) databases to assess the relationships between N6-methyladenosine (m^6^A)-related genes and ACC.

We used the Wilcoxon signed-rank test to compare m^6^A-related gene expression in ACC tissues with that in normal tissues. Then, ACC patients were grouped based on a cluster analysis of m^6^A-related gene expression. m^6^A-related genes that were significantly associated with survival were incorporated into a risk signature, and 2 groups were divided according to median risk score. Fisher exact tests were utilized to analyze differences in clinical variables between groups. We compared the overall survival (OS) rates of the groups by means of Kaplan–Meier curves and Cox regression analyses.

We found that RBM15, ZC3H3, YTDHF1, YTDHF2, and ALBH5 were overexpressed in ACC and that KIAA1429, YTHDC1, HNRNPC, WTAP, METTL3, and FTO were down regulated in ACC. In addition, membership in cluster 2 or the high-risk group was associated with advanced clinical factors and poor prognosis. The univariable and multivariable Cox regression analyses showed that risk score can be considered an independent prognostic factor for ACC.

We found that the expression of m^6^A-related genes could be used as an independent prognostic factor in ACC. However, the current study has some limitations, and further studies of m^6^A-related genes in ACC are needed.

## 1 Introduction

Adrenocortical carcinoma (ACC) is considered a rare cancer, having an estimated annual incidence of 2 per one million people.^[1]^ However, the invasiveness of ACC is high, being second only to anaplastic thyroid cancer in endocrine carcinoma, and ACC patients with metastasis have poor prognosis.^[1–3]^ The median overall survival (OS) and 5-year survival rate of ACC patients are 3.21 years and 15% to 44%, respectively.^[4]^ ACC exhibits a bimodal age distribution, with the first peak occurring at 5 years old and the second peak occurring at 40 to 60 years old.^[5]^ Radical resection is considered the first-line treatment for ACC. In recent years, many hub genes in ACC have been identified and confirmed to be associated with prognosis, suggesting their potential as therapeutic targets.^[6–8]^

Much research has been conducted on N6-methyladenosine (m^6^A), the most common RNA modification in eukaryotic mRNAs and lncRNAs.^[9]^ M^6^A methylation is associated with multiple RNA biofunctions and is dynamically regulated via a variety of genes known as “writers”, “erasers,” and “readers”.^[10]^ The “writers” (methyltransferases) catalyze m^6^A modification and upregulate the levels of m^6^A methylation. These genes include Wilms tumor 1-associated protein (WTAP), methyltransferase-like 3 (METTL3), vir like m^6^A methyltransferase associated (KIAA1429/VIRMA), zinc finger CCCH-type containing 13 (ZC3H13), RNA binding motif protein 15 (RBM15) and methyltransferase-like 14 (METTL14).^[11]^ “Erasers” (demethylases) include fat mass and obesity-associated protein (FTO) and alkB homolog 5 (ALKBH5), which downregulate the level of m^6^A methylation.^[12]^ ”Readers” recognize methylation sites, bind to the RNA and carry out biological functions,^[13]^ they include YTH N6-methyladenosine RNA binding proteins 1 and 2 (YTHDF1 and YTHDF2), YTH domain containing 1 and 2 (YTHDC1 and YTHDC2), and heterogeneous nuclear ribonucleoprotein C (HNRNPC).^[14]^

An increasing number of studies have suggested that the m^6^A modification plays a vital role in various malignancies. For example, the upregulation of METTL3 has been found to inhibit the proliferation, migration and invasion of colorectal cancer cells by p38/ERK pathways^[15]^ and predict poor prognosis in hepatocellular carcinoma.^[16]^ In cervical squamous cell carcinoma, FTO expression was found to be upregulated in tumor tissues and to regulate chemoradiotherapy resistance by targeting beta-catenin.^[17]^ However, the relationships between m^6^A-related genes and ACC remain unknown. Hence, we assessed m^6^A-related gene expression in ACC and evaluated the correlations between m^6^A modifications and ACC prognosis using public datasets. Gene expression data and clinical data for ACC were downloaded from The Cancer Genome Atlas (TCGA), and gene expression data for normal adrenal gland tissue were obtained from the Genotype-Tissue Expression (GTEx) databases.

## 2 Materials and methods

### 2.1 Patient information

We downloaded gene expression and clinical data of ACC patients from TCGA (https://portal.gdc.cancer.gov/). RNA-seq transcriptome data for normal adrenal tissue were obtained from GTEx (https://www.gtexportal.org/). We normalized and merged the transcriptome data from 79 ACC tissues and 127 normal adrenal tissues from the 2 databases into a single dataset. We used the single dataset to screen differentially expressed m^6^A-related genes. Among the 79 ACC tissues, 77 patients had both gene expression and clinical information, which were included for further prognosis analysis. Information on the 77 ACC patients is shown in Table 1. Then, 13 m^6^A-related regulatory genes described in the published literature were selected for analysis (METTL3, METTL14, WTAP, KIAA1429, RBM15, ZC3H13, YTHDC1, YTHDC2, YTHDF1, YTHDF2, HNRNPC, FTO, and ALKBH5).^[10–12]^ Additionally, copy number variation (CNV) data of 90 ACC tissues and 90 normal tissues were also obtained in TCGA database.

**Table 1:** Characteristics of the included ACC patients obtained from the TCGA database.

Basic information	Total (n = 77)	%
Age	48 (median)	
Gender		
Female	48	62.3
Male	29	37.7
Stage		
I	9	11.6
II	37	48.1
III	16	20.8
IV	15	19.5
T classification		
T1	9	11.7
T2	42	54.5
T3	8	10.4
T4	18	23.4
N classification		
N0	68	88.3
N1	9	11.7
M classification		
M0	62	80.5
M1	15	19.5

### 2.2 Bioinformatic analysis

Based on the expression levels of m^6^A-related genes, cluster analysis was performed to cluster the patients of ACC into different groups using the Consensus Cluster Plus package (http://www.bioconductor.org/packages/ConsensusClusterPlus) of R software.^[18]^ We then utilized principal component analysis to observe the patterns of m^6^A-related gene expression in the groups.

To analyze the relationships between m^6^A-related genes and ACC prognosis, we performed univariable Cox regression and identified the genes that were closely related to survival (*P* < .05). Then, the least absolute shrinkage and selection operator (LASSO) Cox regression algorithm was performed to determine coefficients of each selected gene. All Cox regressions were completed via the survival package (https://cran.r-project.org/web/packages/survial) of R software. We used the coefficients to calculate risk scores according to the following formula to build a potential risk signature:

Where xi is the relative expression of genes closely related to survival.^[19]^ The receiver operating characteristic (ROC) curve was utilized to validate the accuracy of risk score using the survival ROC package (https://cran.r-project.org/web/packages/survivalROC) of R software.

### 2.3 Statistical analysis

To compare the expression of m^6^A-related genes in ACC tissues with that in normal tissues, we conducted Wilcoxon signed-rank tests using the limma package (http://www.bioconductor.org/packages/limma) of R. Correlations between m^6^A-related genes were determined using the corrplot package (https://cran.r-project.org/web/packages/corrplot) of R. Then, ACC patients were grouped based on the cluster analysis or risk score (with the median value of risk score considered the cut-off value). Chi-square tests were used to evaluate the differences in CNV between tumor tissues and normal tissues. Fisher exact tests were used to evaluate the differences in clinical variables between clusters and between risk-score groups.

To compare OS between cluster groups or risk groups, Kaplan–Meier survival curves were generated and compared with the log-rank test via the survival package (https://cran.r-project.org/web/packages/survial) of R. Next, we performed univariable and multivariable Cox regression analyses to evaluate whether risk score is an independent predictor of poor OS in ACC patients. Before assessing for multivariable Cox regression, proportional hazard assumption for each covariate was tested via in minus in survival curves. By observing the in minus in survival curves of each covariate in 2 states (Age ≤48 vs Age >48, female vs male, T1&2 vs T3&4, Stage I&II vs Stage III&IV, N0 vs N1 and M0 vs M1), if the 2 survival curves were parallel, indicating that the proportional hazard assumption was true, and the covariates included in the study period had the same impact on survival at any time. These meant that it was appropriate to use the multivariable Cox regression model. We used R 3.5.3 software (https://www.r-project.org/) and SPSS (version 26.0) to perform all statistical analyses.

## 3 Results

### 3.1 m^6^A-related gene expression in ACC and normal tissues

**Figure 1:**
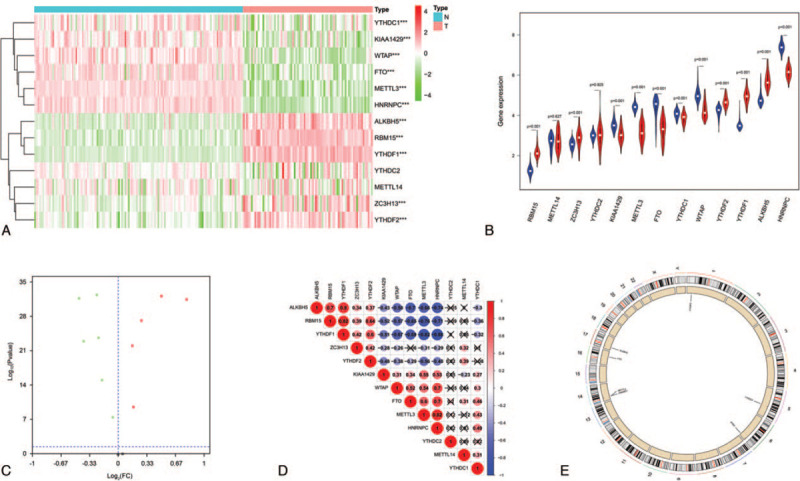
m^6^A-related gene expression in ACC tissues and normal tissues. We used the Wilcoxon signed-rank test to compare the expression of m^6^A-related genes in ACC tissues with that in normal tissues. (A) Heatmap depicting m^6^A-related gene expression in samples ranging from high (red) to low (green). The expression levels of RBM15, ZC3H3, YTDHF1, YTDHF2, and ALBH5 were significantly higher in ACC tissues than in normal tissues (all *P* values <.001). The expression levels of KIAA1429, YTHDC1, HNRNPC, WTAP, METTL3, and FTO were significantly lower in ACC tissues than in normal tissues (all *P* values <.001). (B) Violin plot of the expression of the m^6^A-related genes in ACC tissues (red) and normal adrenal gland tissues (blue). (C) Volcano plot of the expression of the m^6^A-related genes in ACC tissues and normal adrenal gland tissues. Red dots represented high expression genes, green dots represented low expression genes, and black dots represented unchanged genes. (D) Pearson correlation analysis of m^6^A-related genes. The plot depicts the coefficients of correlations between m^6^A-related genes ranging from high (red) to low (blue). (E) The results of CNV analysis showed that compared with normal adrenal gland tissues, YTHDF2 was single deleted on chromosome 1, YTHDC1 and METTL14 were single gained on chromosome 4, YTHDC2 was single gained on chromosome 5, KIAA1429 was single gained on chromosome 8, FTO was single gained on chromosome 16, ALKBH5 was single deleted on chromosome 17, and YTHDF1 was single gained on chromosome 20. ACC = adrenocortical carcinoma, ALKBH5 = alkB homolog 5, FTO = obesity-associated protein, HNRNPC = heterogeneous nuclear ribonucleoprotein C, KIAA1429 = vir like m^6^A methyltransferase associated, M^6^A = N6-methyladenosine, METTL3 and 14 = methyltransferase-like 3 and 14, N = normal, RBM15 = RNA binding motif protein 15, T = tumor, WTAP = Wilms tumor 1-associated protein, YTHDC1 and 2 = YTH domain containing 1 and 2, YTHDF1 and 2 = YTH N6-methyladenosine RNA binding protein 1 and 2, ZC3H13 = zinc finger CCCH-type containing 13; ^∗^, *P* < .05; ^∗∗^, *P* < .01; ^∗∗∗^, *P* < .001.

To compare m^6^A-related gene expression in the 79 ACC tissues with that in the 127 normal tissues, we used the Wilcoxon signed-rank test. RBM15, ZC3H3, YTDHF1, YTDHF2 and ALBH5 were overexpressed in ACC tissues relative to normal tissues (all *P* values <.001). The expression levels of KIAA1429, YTHDC1, HNRNPC, WTAP, METTL3, and FTO were downregulated in ACC tissues (all *P* values <.001). All the results were shown by heatmap, violin plot and volcano plot. (Fig. 1A-C). We then explored the correlations between m^6^A-related genes. Among all pairwise combinations of m^6^A-related genes, HNRNPC and METTL3 had the highest positive correlation of expression, and RBM15 and METTL3 had the highest negative correlation (Fig. 1D). The results of CNV analysis showed that compared with normal adrenal gland tissues, YTHDF2 was single deleted on chromosome 1, YTHDC1 and METTL14 were single gained on chromosome 4, YTHDC2 was single gained on chromosome 5, KIAA1429 was single gained on chromosome 8, FTO was single gained on chromosome 16, ALKBH5 was single deleted on chromosome 17, and YTHDF1 was single gained on chromosome 20 (Fig. 1E).

### 3.2 Grouping based on the cluster analysis was significantly correlated with prognosis

**Figure 2:**
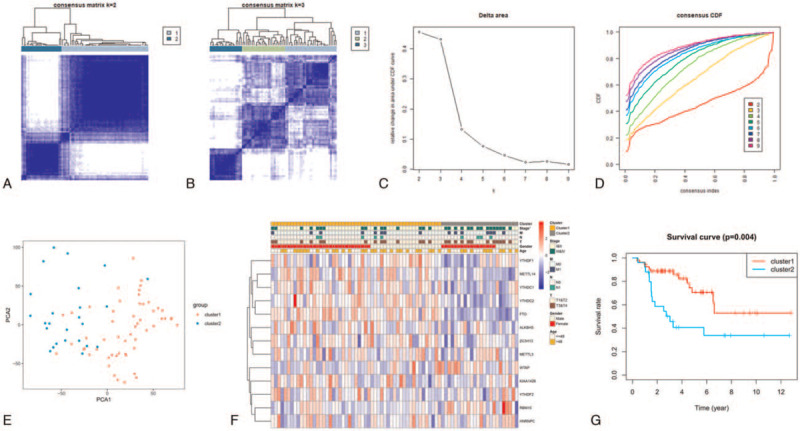
Cluster analysis results and prognosis of ACC patients in the TCGA database. (A) Consensus clustering matrix for k = 2. The plot depicts consensus values on a white to blue color scale. (B) Consensus clustering matrix for k = 3. A-B present intra- and intergroup consensus clustering into k groups (C) Relative change in area under the CDF curve for k = 2 to 9. (D) Consensus clustering CDF for k = 2 to 9. C-D show that from k = 3 to 4, the relative change of CDF was low. This result suggested that k = 3 was appropriate; however, as shown in B, k = 3 yielded low consensus. Thus, k = 2, with high consensus, was selected; results are shown in A. (E) PCA of cluster 1 and cluster 2, revealing unambiguous distribution of between cluster 1 and cluster 2. (F) Heatmap showing significant differences in advanced clinical stage between cluster 1 and cluster 2. (G) Kaplan–Meier survival curves showing that the prognosis of cluster 2 was worse than that of cluster 1. ACC = adrenocortical carcinoma, CDF = cumulative distribution function, PCA = principal component analysis, TCGA = The Cancer Genome Atlas ^∗^, *P* < .05.

We used cluster analysis to cluster the ACC patients into different groups based on m^6^A-related gene expression (Fig. 2A-D), ultimately dividing them into 2 groups: cluster 1 and cluster 2. Principal component analysis was utilized to observe the patterns of m^6^A-related gene expression in the 2 groups (Fig. 2E), which were unambiguously distributed between cluster 1 and cluster 2. Fisher exact tests showed that cluster 2 was significantly associated with advanced clinical stage (Fig. 2F) (Table 2). Additionally, the Kaplan–Meier survival curves showed that the prognosis of patients with ACC in cluster 2 was worse than that of patients in cluster 1 (*P* = .004) (Fig. 2G).

**Table 2:** Differences in the characteristics of the ACC patients in cluster 1 and cluster 2.

Basic information	Cluster 1	Cluster 2	*P* value
Total	53	24	
Age			.628
≤48	25	13	
> 48	28	11	
Gender			.324
Female	31	17	
Male	22	7	
Stage			.011
I&II	37	9	
III&IV	16	15	
T classification			.067
T1&T2	39	12	
T3&T4	14	12	
N classification			.448
N0	48	20	
N1	5	4	
M classification			.213
M0	45	17	
M1	8	7	

### 3.3 Grouping based on risk score was strongly associated with prognosis

**Figure 3:**
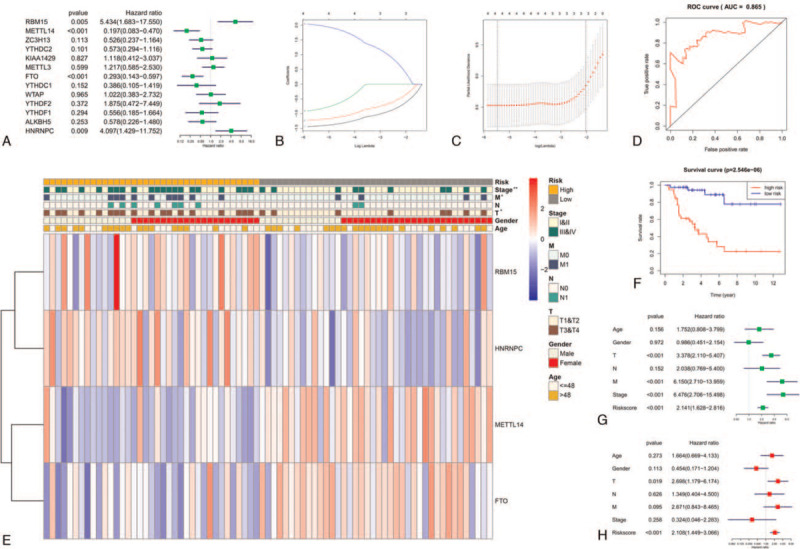
Risk scores and prognosis of ACC patients in the TCGA database. (A) Four genes, namely, HNRNPC, RBM15, METTL14 and FTO, were selected to construct a risk signature using univariable Cox regression (*P* < .05). (B-C) Risk score was calculated based on the coefficients obtained from LASSO Cox regression. (D) ROC curve showing that the OS of the ACC patients was perfectly predicted by risk score. (E) Heatmap showing significant differences in advanced clinical stage, high M classification, and high T classification between the high-risk group and the low-risk group. (F) Kaplan–Meier survival curves revealing that the OS of patients with ACC in the high-risk group was worse than that of patients with ACC in the low-risk group. (G) Univariable Cox regression revealed that clinical stage, high M classification, high T classification, advanced clinical stage and high risk score were strongly correlated with poor prognosis (*P* < .05). (H) Multivariable Cox regression identified T classification and risk score as associated with prognosis (*P* < .05). ACC = adrenocortical carcinoma, FTO = obesity-associated protein, HNRNPC = heterogeneous nuclear ribonucleoprotein C, LASSO = least absolute shrinkage and selection operator, METTL14 = methyltransferase-like 14, RBM15 = RNA binding motif protein 15, ROC = receiver operating characteristic, TCGA = The Cancer Genome Atlas ^∗^, *P* < .05; ^∗∗^, *P* < .01.

We used univariable Cox regression to analyze the relationship between the OS of ACC patients and the expression of m^6^A-related genes (Fig. 3A). The expression levels of 4 genes, namely, HNRNPC, RBM15, METTL14, and FTO, were found to be significantly associated with prognosis. We then applied LASSO Cox regression to establish gene coefficients. The results showed that the risk signature could be constructed using all 4 genes, and risk scores were calculated based on the coefficients from the LASSO Cox regression (Fig. 3B-C). Two groups of patients were established according to median risk score. The ROC curve results showed that the OS of ACC patients was perfectly predicted by risk score (AUC = 0.865) (Fig. 3D). The Fisher exact tests revealed that membership in the high-risk group of ACC patients was significantly associated with advanced clinical stage, high M classification and high T classification (Fig. 3E) (Table 3). The Kaplan–Meier survival curves revealed that membership in the high-risk group of ACC patients implied poor prognosis (*P* < .001) (Fig. 3F). Univariable Cox regression showed that high M classification, high T classification, advanced clinical stage and high risk score were strongly correlated with poor prognosis (Fig. 3G). We then performed multivariable Cox regression, which identified risk score and T classification were associated with prognosis (Fig. 3H). This result indicates that a high risk score is an independent predictor of poor OS in ACC. Before assessing for multivariable Cox regression, the 2 survival curves of each covariate were parallel, indicating that the proportional hazard assumption was true (Supplementary Fig. 1A-G).

**Table 3:** Differences in the characteristics of ACC patients between the high risk and low risk.

Basic information	Low risk	High risk	*P* value
Total	40	37	
Age			1.000
≤48	20	18	
>48	20	17	
Gender			1.000
Female	25	23	
Male	15	14	
Stage			.001
I&II	31	15	
III&IV	9	22	
T classification			.003
T1&T2	7	19	
T3&T4	38	18	
N classification			.079
N0	38	30	
N1	2	7	
M classification			.008
M0	37	25	
M1	3	12	

## 4 Discussion

With the development of epigenetics, m^6^A methylation has become a promising research direction and has been demonstrated to be closely associated with the occurrence and development of tumors.^[20–23]^ The overexpression of a “writer” of m^6^A, METTL3, was correlated with the poor prognosis of bladder carcinoma by positively modulating pri-miR221/222.^[24]^ In pancreatic cancer cells, the knockdown of METTL3 was found to decrease chemo- and radio-resistance,^[25]^ and increased expression of METTL3 was found to promote the invasion and migration of melanoma cells via the actions of matrix metallopeptidase 2.^[26]^ A “reader” of m^6^A, YTHDF1, was found to be overexpressed in colorectal cancer and to stimulate the invasion of colorectal cancer cells by activating the Wnt/beta-catenin pathway.^[27]^ In acute myeloid leukemia, the upregulation of YTHDF2 was found to decrease the half-life of diverse m^6^A transcripts, and YTHDF2 knockdown was shown to inhibit cancer stem cell development.^[28]^ Furthermore, an “eraser” of m^6^A, FTO, was shown to be suppressed in clear cell renal cell carcinoma (ccRCC) tissues, and overexpression of FTO was found to activate oxidative stress and ROS production by increasing the expression of PGC-1α.^[29]^ Although an increasing number of studies have confirmed that m^6^A modification is significantly associated with tumors, the relationships between m^6^A modification and ACC have to date been unknown.

Here, we compared the expression of m^6^A-related genes between ACC tissues and normal adrenal gland tissues. In ACC relative to normal tissue, RBM15, ZC3H3, YTDHF1, YTDHF2, and ALBH5 were overexpressed, and the expression levels of KIAA1429, YTHDC1, HNRNPC, WTAP, METTL3, and FTO in ACC were low. Based on the cluster analysis results, the ACC patients were grouped into clusters 1 and 2. The prognosis of cluster 2, which was associated with advanced clinical stage and high T classification, was worse than that of cluster 1. We constructed a risk signature through LASSO Cox regression based on 4 m^6^A genes identified as associated with prognosis by univariable Cox regression and calculated risk scores. The prognosis of the high-risk group, which was significantly related to advanced clinical stage, higher T classification and M classification, was worse than that of the low-risk group. The univariable and multivariable Cox regression results showed that the risk score of m^6^A modification can be used as an independent prognostic factor in ACC. Taken together, our results demonstrate that the expression of m^6^A-related genes is closely related to clinical variables and prognosis in ACC. These results indicate that expression of m^6^A-related genes can serve as an independent prognostic factor in ACC and that such genes offer potential new drug targets in ACC, as targeting m^6^A may be an effective treatment for tumors.^[30]^

The biological functions of m^6^A related genes should also be focused. M^6^A methyltransferase complex (“writers”) could mediate m^6^A methylation of RNAs, a modification that plays a role in the efficiency of mRNA splicing and RNA processing. Previous studies have found that METTL3 and METTL14 formed stable heterodimers, which interacted with WTP to constitute WMM complex (WTP- METTL3- METTL14), which promoted the deposition of m^6^A.^[31]^ Recent in-depth studies indicated that KIAA1429 led to the preferential mRNA methylation in 3′UTR and near the stop codon, then recruited WMM as the core to guide m^6^A methylation at specific sites.^[32]^ As an RNA binding protein, RBM15 was involved in the regulation of hematopoietic cell homeostasis and mRNA alternative splicing and recruited WMM complex to specific RNA sites during the m^6^A methylation.^[33]^ ZC3H13 was considered to be a component of WMM complex, which could improve the catalytic function of WMM complex by interacting with WTAP.^[34,35]^ As demethylase, the “erasers”(FTO and ALKBH5) used ferrous as cofactor, and α-ketoglutarate was used as cosubstrate to remove the methylation of m^6^A.^[36]^ FTO promotes adipocyte cycle progression and adipogenesis by reducing the m^6^A level of cyclin A2 and cyclin dependent kinase 2, and controls mRNA splicing by inhibiting serine and arginine rich splicing factor 2 binding at splicing sites.^[37,38]^ Demethylation induced by ALKBH5 was related to splicing and the production of long 3 ’-UTR mRNA.^[39,40]^ As “readers” of m^6^A methylation (YTHDC1, YTHDC2, YTHDF1, YTHDF2 and HNRNPC), the function was to recognize and bind to the m^6^A site.^[41,42]^ YTHDF1 could promote the synthesis of protein and translation of mRNA.^[43]^ By binding to m^6^A-modified mRNA, YTHDF2 activated transcription product degradation.^[44]^ YTHDC1 was significantly associated with RNA splicing and export.^[45,46]^ YTHDC2 enhanced the target RNAs translation efficiency, but decreased their abundance.^[47]^ HNRNPC was involved in regulating mRNA splicing.^[48]^

The current study has some limitations. The number of ACC tissues was low, and paired normal tissue controls were not available in the TCGA database; thus, we had to pool the data from 2 databases. In addition, the protein levels of m^6^A-related genes in ACC could not be reliably estimated; thus, we focused on the relationship between m^6^A-related gene expression and ACC prognosis. Furthermore, the biological functions and mechanisms of the m^6^A-related genes were not explored. Moreover, previous studies have shown that other genes with abnormal expression in ACC can affect prognosis in ACC. For example, the upregulation of urothelial carcinoma-associated 1 (UCA1) promoted the proliferation and inhibited the apoptosis of ACC cells though the miR-298-CDK6 axis.^[49]^ Overexpression of HOX transcript antisense RNA (HOTAIR), by regulating the cell cycle, led to the poor OS in ACC.^[50]^ High expression of topoisomerase alpha 2 (TOP2A) was demonstrated to activate ACC cell proliferation, with TOP2A representing a potential therapeutic target.^[51]^ We tried to explore the relationships between m^6^A-related genes and these ACC-driver genes. But the relationships could not be clearly determined due to a lack of studies. Moreover, because the mechanisms of m^6^A regulating the development of tumors were very complex, we admitted that the researches on the related mechanisms were not deep enough, and no basic experiment were performed, which also became a limitation of our article. Therefore, further studies of m^6^A-related genes in ACC are needed.

## 5 Conclusions

In the current study, we compared m^6^A-related gene expression between ACC tissues and normal adrenal gland tissues. We found that the expression of m^6^A-related genes could be used as an independent prognostic factor in ACC. However, the current study has some limitations, and further studies of m^6^A-related genes in ACC are needed.

## Author contributions

**Conceptualization:** Chuize Kong and Lei Yin.

**Data curation:** Yang Fu, Shanshan Sun.

**Formal analysis:** Yang Fu, Shanshan Sun, Jianbin Bi.

**Funding acquisition:** Chuize Kong.

**Methodology:** Yang Fu, Lei Yin.

**Project administration:** Yang Fu, Jianbin Bi.

**Resources:** Jianbin Bi, Chuize Kong.

**Software:** Yang Fu, Shanshan Sun.

**Supervision:** Yang Fu.

**Writing – original draft:** Yang Fu, Shanshan Sun.

**Writing – review & editing:** Jianbin Bi, Chuize Kong, Lei Yin.

## Supplementary Material

Supplemental Digital Content

## References

[1] Bilimoria KY Shen WT Elaraj D. Adrenocortical carcinoma in the United States: treatment utilization and prognostic factors. Cancer 2008;113:3130–6. 18973179 10.1002/cncr.23886

[2] Xiao H He W Chen P. Identification of seven aberrantly methylated and expressed genes in adrenocortical carcinoma. Front Endocrinol (Lausanne) 2019;10:472–87. 31354635 10.3389/fendo.2019.00472 PMC6640086

[3] Schteingart DE Doherty GM Gauger PG. Management of patients with adrenal cancer: recommendations of an international consensus conference. Endocr Relat Cancer 2005;12:667–80. 16172199 10.1677/erc.1.01029

[4] Tella SH Kommalapati A Yaturu S. Predictors of survival in adrenocortical carcinoma: an analysis from the national cancer database. J Clin Endocrinol Metab 2018;103:3566–73. 29982685 10.1210/jc.2018-00918

[5] Fernandez Ranvier GG Inabnet WB. Surgical management of adrenocortical carcinoma. Endocrinol Metab Clin North Am 2015;44:435–52. 26038210 10.1016/j.ecl.2015.02.008

[6] Yuan L Qian G Chen L. Co-expression network analysis of biomarkers for adrenocortical carcinoma. Front Genet 2018;9:328–40. 30158955 10.3389/fgene.2018.00328 PMC6104177

[7] Romero Arenas MA Whitsett TG Aronova A. Protein expression of PTTG1 as a diagnostic biomarker in adrenocortical carcinoma. Ann Surg Oncol 2018;25:801–7. 29218429 10.1245/s10434-017-6297-1

[8] Liang J Liu Z Wei X. Expression of FSCN1 and FOXM1 are associated with poor prognosis of adrenocortical carcinoma patients. BMC Cancer 2019;19:1165–73. 31783819 10.1186/s12885-019-6389-3 PMC6884893

[9] Lobo J Barros-Silva D Henrique R. The emerging role of epitranscriptomics in cancer: focus on urological tumors. Genes (Basel) 2018;9:552–72. 10.3390/genes9110552 PMC6265908 30428628

[10] Zhou J Wang J Hong B. Gene signatures and prognostic values of m6A regulators in clear cell renal cell carcinoma - a retrospective study using TCGA database. Aging (Albany NY) 2019;11:1633–47. 30877265 10.18632/aging.101856 PMC6461179

[11] Yang Y Hsu PJ Chen YS. Dynamic transcriptomic m (6)A decoration: writers, erasers, readers and functions in RNA metabolism. Cell Res 2018;28:616–24. 29789545 10.1038/s41422-018-0040-8 PMC5993786

[12] Zhang C Zhang M Ge S. Reduced m6A modification predicts malignant phenotypes and augmented Wnt/PI3K-Akt signaling in gastric cancer. Cancer Med 2019;8:4766–81. 31243897 10.1002/cam4.2360 PMC6712480

[13] Tuncel G Kalkan R. Importance of m N6-methyladenosine (m6A) RNA modification in cancer. Med Oncol 2019;36:36–41. 30879160 10.1007/s12032-019-1260-6

[14] Chai R-C Wu F Wang Q-X. m^6^A RNA methylation regulators contribute to malignant progression and have clinical prognostic impact in gliomas. Aging 2019;11:1204–25. 30810537 10.18632/aging.101829 PMC6402513

[15] Deng R Cheng Y Ye S. m(6)A methyltransferase METTL3 suppresses colorectal cancer proliferation and migration through p38/ERK pathways. Onco Targets Ther 2019;12:4391–402. 31239708 10.2147/OTT.S201052 PMC6556107

[16] Chen M Wei L Law CT. RNA N6-methyladenosine methyltransferase-like 3 promotes liver cancer progression through YTHDF2-dependent posttranscriptional silencing of SOCS2. Hepatology 2018;67:2254–70. 29171881 10.1002/hep.29683

[17] Zhou S Bai ZL Xia D. FTO regulates the chemo-radiotherapy resistance of cervical squamous cell carcinoma (CSCC) by targeting beta-catenin through mRNA demethylation. Mol Carcinog 2018;57:590–7. 29315835 10.1002/mc.22782

[18] Wilkerson MD Hayes DN. ConsensusClusterPlus: a class discovery tool with confidence assessments and item tracking. Bioinformatics (Oxford, England) 2010;26:1572–3. 10.1093/bioinformatics/btq170 PMC2881355 20427518

[19] Wang Z Song Q Yang Z. Construction of immune-related risk signature for renal papillary cell carcinoma. Cancer Med 2019;8:289–304. 30516029 10.1002/cam4.1905 PMC6346237

[20] Zhu Z Qian Q Zhao X. N(6)-methyladenosine (m(6)A) ALKBH5 promotes the non-small cell lung cancer progress by regulating TIMP3 stability. Gene 2020;731:144348–64. 31927006 10.1016/j.gene.2020.144348

[21] Liu L Wang J Sun G. m(6)A mRNA methylation regulates CTNNB1 to promote the proliferation of hepatoblastoma. Mol Cancer 2019;18:188–200. 31870368 10.1186/s12943-019-1119-7 PMC6927193

[22] Cai J Yang F Zhan H. RNA m(6)A methyltransferase METTL3 promotes the growth of prostate cancer by regulating hedgehog pathway. Onco Targets Ther 2019;12:9143–52. 31806999 10.2147/OTT.S226796 PMC6842310

[23] Liu T Yang S Sui J. Dysregulated N6-methyladenosine methylation writer METTL3 contributes to the proliferation and migration of gastric cancer. J Cell Physiol 2020;235:548–62. 31232471 10.1002/jcp.28994

[24] Han J Wang JZ Yang X. METTL3 promote tumor proliferation of bladder cancer by accelerating pri-miR221/222 maturation in m6Adependent manner. Mol Cancer 2019;18:110–24. 31228940 10.1186/s12943-019-1036-9 PMC6588935

[25] Taketo K Konno M Asai A. The epitranscriptome m6A writer METTL3 promotes chemo- and radioresistance in pancreatic cancer cells. Int J Oncol 2018;52:621–9. 29345285 10.3892/ijo.2017.4219

[26] Dahal U Le K Gupta M. RNA m6A methyltransferase METTL3 regulates invasiveness of melanoma cells by matrix metallopeptidase 2. Melanoma Res 2019;29:382–9. 30762711 10.1097/CMR.0000000000000580

[27] Bai Y Yang C Wu R. YTHDF1 regulates tumorigenicity and cancer stem cell-like activity in human colorectal carcinoma. Front Oncol 2019;9:332–43. 31131257 10.3389/fonc.2019.00332 PMC6509179

[28] Paris J Morgan M Campos J. Targeting the RNA m(6)A reader YTHDF2 selectively compromises cancer stem cells in acute myeloid leukemia. Cell Stem Cell 2019;25:137–48. e136. 31031138 10.1016/j.stem.2019.03.021 PMC6617387

[29] Zhuang C Zhuang C Luo X. N6-methyladenosine demethylase FTO suppresses clear cell renal cell carcinoma through a novel FTO-PGC-1alpha signalling axis. J Cell Mol Med 2019;23:2163–73. 30648791 10.1111/jcmm.14128 PMC6378205

[30] Deng X Su R Weng H. RNA N(6)-methyladenosine modification in cancers: current status and perspectives. Cell Res 2018;28:507–17. 29686311 10.1038/s41422-018-0034-6 PMC5951805

[31] Liu J Yue Y Han D. A METTL3-METTL14 complex mediates mammalian nuclear RNA N6-adenosine methylation. Nat Chem Biol 2014;10:93–5. 24316715 10.1038/nchembio.1432 PMC3911877

[32] Yue Y Liu J Cui X. VIRMA mediates preferential m(6)A mRNA methylation in 3’UTR and near stop codon and associates with alternative polyadenylation. Cell Discovery 2018;4:10–26. 29507755 10.1038/s41421-018-0019-0 PMC5826926

[33] Patil DP Chen CK Pickering BF. m(6)A RNA methylation promotes XIST-mediated transcriptional repression. Nature 2016;537:369–73. 27602518 10.1038/nature19342 PMC5509218

[34] Wen J Lv R Ma H. Zc3h13 regulates nuclear RNA m(6)A methylation and mouse embryonic stem cell self-renewal. Mol Cell 2018;69:1028–38. e1026. 29547716 10.1016/j.molcel.2018.02.015 PMC5858226

[35] Knuckles P Lence T Haussmann IU. Zc3h13/Flacc is required for adenosine methylation by bridging the mRNA-binding factor Rbm15/Spenito to the m(6)A machinery component Wtap/Fl(2)d. Genes Development 2018;32:415–29. 29535189 10.1101/gad.309146.117 PMC5900714

[36] Fedeles BI Singh V Delaney JC. The AlkB family of Fe(II)/α-Ketoglutarate-dependent dioxygenases: repairing nucleic acid alkylation damage and beyond. J Biological Chem 2015;290:20734–42. 10.1074/jbc.R115.656462 PMC4543635 26152727

[37] Zhao X Yang Y Sun BF. FTO-dependent demethylation of N6-methyladenosine regulates mRNA splicing and is required for adipogenesis. Cell Res 2014;24:1403–19. 25412662 10.1038/cr.2014.151 PMC4260349

[38] Wu R Liu Y Yao Y. FTO regulates adipogenesis by controlling cell cycle progression via m(6)A-YTHDF2 dependent mechanism. Biochimica et Biophysica Acta Mol Cell Biol Lipids 2018;1863:1323–30. 10.1016/j.bbalip.2018.08.008 30305247

[39] Zheng G Dahl JA Niu Y. ALKBH5 is a mammalian RNA demethylase that impacts RNA metabolism and mouse fertility. Mol Cell 2013;49:18–29. 23177736 10.1016/j.molcel.2012.10.015 PMC3646334

[40] Tang C Klukovich R Peng H. ALKBH5-dependent m^6^A demethylation controls splicing and stability of long 3’-UTR mRNAs in male germ cells. Proc Natl Acad Sci U S A 2018;115:e325–33. 29279410 10.1073/pnas.1717794115 PMC5777073

[41] Chen XY Zhang J Zhu JS. The role of m(6)A RNA methylation in human cancer. Mol Cancer 2019;18:103–11. 31142332 10.1186/s12943-019-1033-z PMC6540575

[42] Casella G Tsitsipatis D Abdelmohsen K. mRNA methylation in cell senescence. Wiley Interdiscip Rev RNA 2019;10:e1547–58. 31144457 10.1002/wrna.1547 PMC8474013

[43] Wang X Zhao BS Roundtree IA. N(6)-methyladenosine modulates messenger RNA translation efficiency. Cell 2015;161:1388–99. 26046440 10.1016/j.cell.2015.05.014 PMC4825696

[44] Wang X Lu Z Gomez A. N6-methyladenosine-dependent regulation of messenger RNA stability. Nature 2014;505:117–20. 24284625 10.1038/nature12730 PMC3877715

[45] Roundtree IA Luo GZ Zhang Z. YTHDC1 mediates nuclear export of N(6)-methyladenosine methylated mRNAs. eLife 2017;6:e31311–37. 28984244 10.7554/eLife.31311 PMC5648532

[46] Kasowitz SD Ma J Anderson SJ. Nuclear m6A reader YTHDC1 regulates alternative polyadenylation and splicing during mouse oocyte development. PLoS Genet 2018;14:e1007412–39. 29799838 10.1371/journal.pgen.1007412 PMC5991768

[47] Hsu PJ Zhu Y Ma H. Ythdc2 is an N(6)-methyladenosine binding protein that regulates mammalian spermatogenesis. Cell Res 2017;27:1115–27. 28809393 10.1038/cr.2017.99 PMC5587856

[48] Liu N Dai Q Zheng G. N(6)-methyladenosine-dependent RNA structural switches regulate RNA-protein interactions. Nature 2015;518:560–4. 25719671 10.1038/nature14234 PMC4355918

[49] Guo N Sun Q Fu D. Long non-coding RNA UCA1 promoted the growth of adrenocortical cancer cells via modulating the miR-298-CDK6 axis. Gene 2019;703:26–34. 30935924 10.1016/j.gene.2019.03.066

[50] Yan ZC He L Qiu JH. LncRNA HOTAIR participates in the development and progression of adrenocortical carcinoma via regulating cell cycle. Eur Rev Med Pharmacol Sci 2018;22:6640–9. 30402836 10.26355/eurrev_201810_16139

[51] Jain M Zhang L He M. TOP2A is overexpressed and is a therapeutic target for adrenocortical carcinoma. Endocr Relat Cancer 2013;20:361–70. 23533247 10.1530/ERC-12-0403 PMC4990817

