# Supplementary material for: Expression patterns and prognostic value of m6A RNA methylation regulators in adrenocortical carcinoma
Source: Medicine (Baltimore). 2021 Mar 12;100(10):e25031. doi: 10.1097/MD.0000000000025031 (PMC7969304; doi:10.1097/MD.0000000000025031)
Supplement: Supplemental Digital Content [file medi-100-e25031-s001.doc]

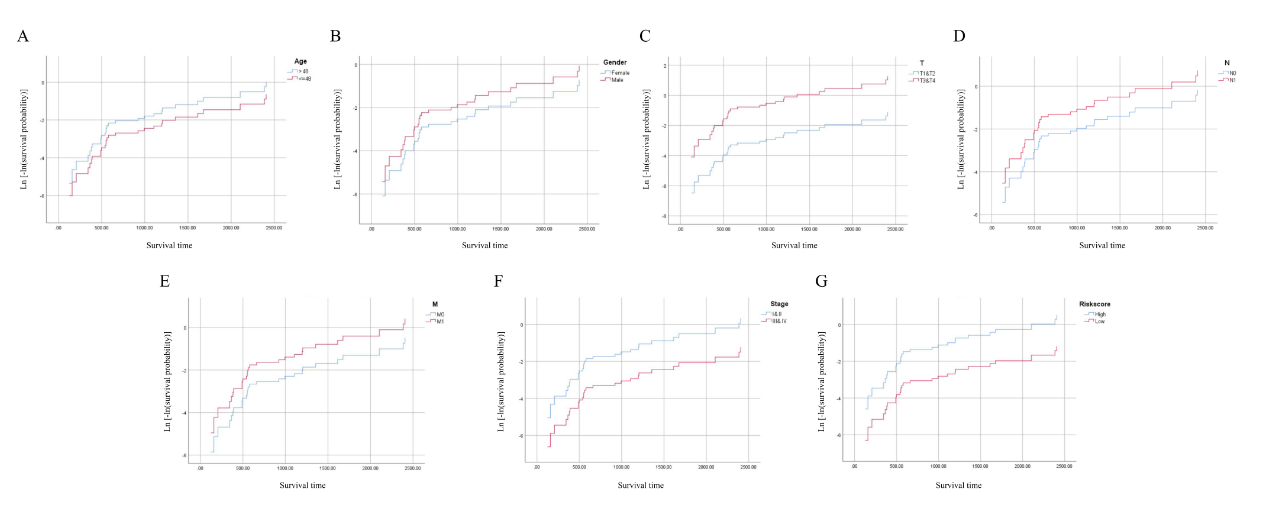


**Supplementary Figure 1.** The proportional hazard assumption was performed for each covariate via ln minus ln survival curves, including age (A), gender (B), T classification (C), N classification (D), M classification (E), clinical stage (F) and riskscore (G).
